# Supplementary material for: Interior Hotspot Engineering in Ag–Au Bimetallic Nanocomposites by In Situ Galvanic Replacement Reaction for Rapid and Sensitive Surface-Enhanced Raman Spectroscopy Detection
Source: Int J Mol Sci. 2022 Oct 3;23(19):11741. doi: 10.3390/ijms231911741 (PMC9569599; doi:10.3390/ijms231911741)
Supplement: Supplementary file 1 [file ijms-23-11741-s001.zip › ijms-1880972-supplementary.pdf]

International Journal of
Molecular Sciences

MDPI

Supplementary Material

Interior hotspot engineering in Ag–Au bimetallic nanocomposites by *in situ* galvanic replacement reaction for rapid and sensitive surface-enhanced Raman spectroscopy detection

Iris Baffour Ansah [1,2], Soo Hyun Lee [1], ChaeWon Mun [1], Dong-Ho Kim [1,2] and Sung-Gyu Park [1,*]

[1] Department of Nano-Bio Convergence, Korea Institute of Materials Science, 797 Changwondae-ro, Changwon, 51508, Republic of Korea

[2] Advanced Materials Engineering Division, University of Science and Technology (UST), 217 Gageong-ro, Yuseong-gu, Daejeon 34113, Republic of Korea

* Correspondence: sgpark@kims.re.kr

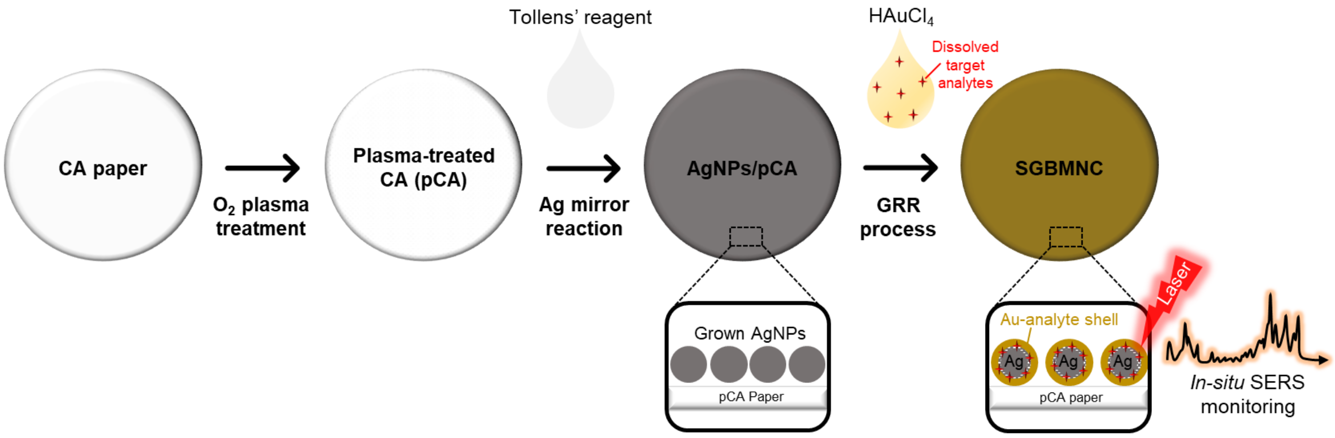

Figure S1. Schematic of the fabrication procedures for the SGBMNC SERS platform *via* chemical syntheses.

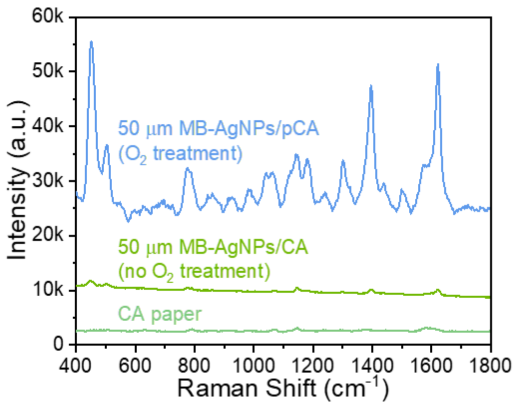

Figure S2. SERS spectra of the bare CA paper and the MB-attached AgNPs grown on the CA with and without oxygen plasma treatment.

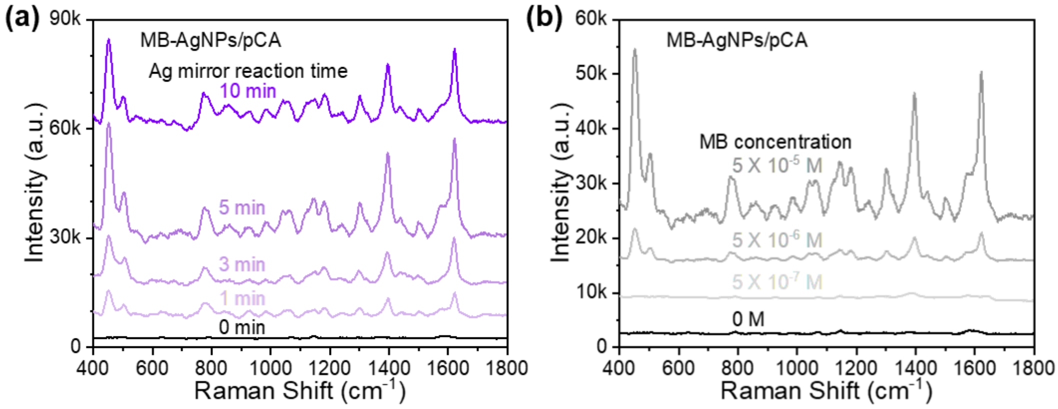

Figure S3. Influence of (a) the Ag mirror reaction time and (b) the molecular concentration on the SERS spectra of MB-AgNPs/pCA.

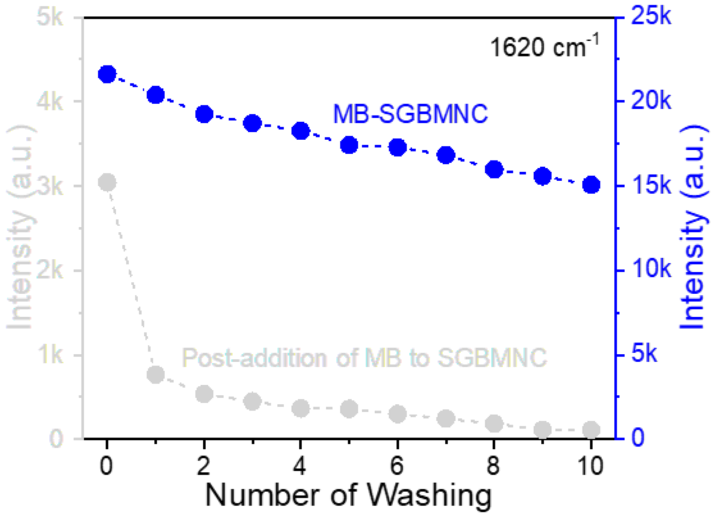

Figure S4. Signal variations of the post-addition of MB to SGBMNC and MB-SGBMNC during ten times washing.

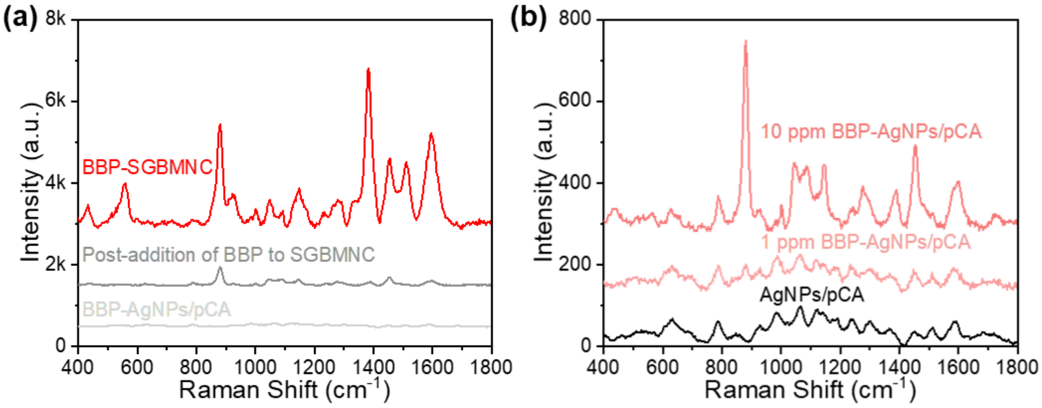

Figure S5. (a) SERS spectra of BBP on the platform with and without interior hotspots and (b) the sensitivity of AgNPs/pCA for BBP detection.
